# Supplementary material for: Comparison of Cardiovascular Risk and Events among Spanish Patients with and without Ocular Pseudoexfoliation
Source: J Clin Med. 2022 Apr 12;11(8):2153. doi: 10.3390/jcm11082153 (PMC9024962; doi:10.3390/jcm11082153)
Supplement: Supplementary file 1 [file jcm-11-02153-s001.zip › jcm-1660690-supplementary.pdf]

AGE			Men without antihypertensive treatment							
			Non-Diabetic							
			Non-smokers				Smokers			
	Cholesterol		<5,2	5,2-6,4	6,5-7,7	≥7,8	<5,2	5,2-6,4	6,5-7,7	≥7,8
>80	SBP	≥180	48	47	48	45	56	57	58	55
		160-180	44	45	46	43	54	54	56	52
		140-160	41	42	44	40	51	51	53	49
		<140	30	30	32	30	38	38	40	36
70-79	SBP	≥180	27	28	29	27	35	35	37	34
		160-180	26	26	28	25	33	33	35	32
		140-160	24	25	26	23	31	31	33	30
		<140	17	17	18	16	22	22	23	21
60-69	SBP	≥180	18	19	19	18	24	24	25	23
		160-180	17	18	18	17	22	23	24	22
		140-160	16	16	17	15	21	21	22	20
		<140	11	11	12	11	14	15	15	15
50-59	SBP	≥180	9	10	10	10	12	12	13	12
		160-180	9	9	9	9	12	12	12	11
		140-160	8	8	9	8	11	11	11	10
		<140	6	6	6	5	7	7	8	7
40-49	SBP	≥180	3	3	4	3	4	5	5	5
		160-180	3	3	3	3	4	4	5	5
		140-160	3	3	3	3	4	4	4	4
		<140	2	2	2	2	3	3	3	3
30-39	SBP	≥180	2	2	2	2	2	2	3	2
		160-180	2	2	2	2	2	2	2	2
		140-160	2	2	2	2	2	2	2	2
		<140	1	1	1	1	1	1	1	1

	Low: <5%
	Mild: 5-9%
	Moderate:10-14%
	Moderate-high: 15-19%
	High: 20-29%
	Very high: ≥30%

Figure S1. ERICE cardiovascular risk chart.

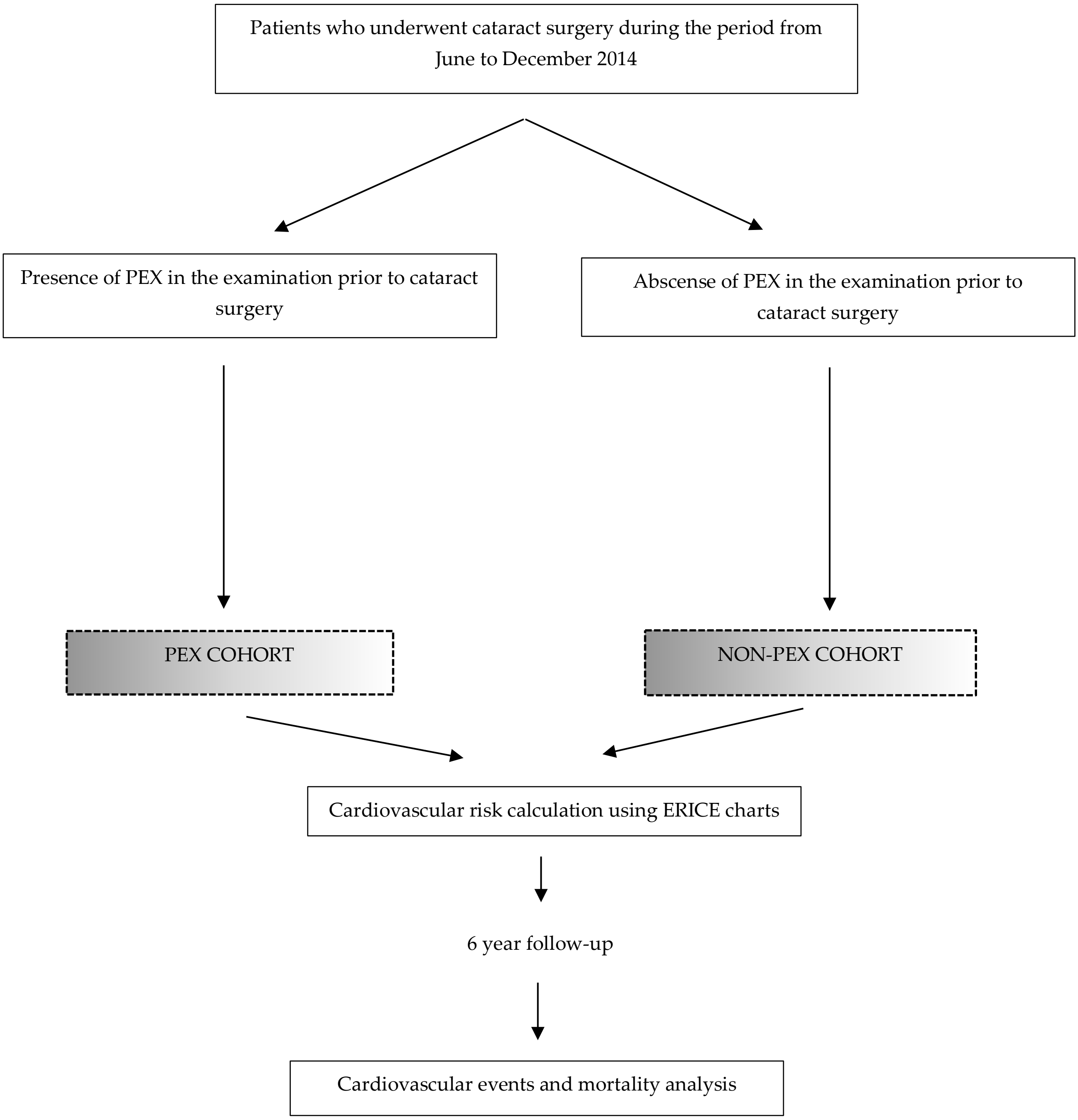

Figure S2. Study flowchart.
